# Whey protein supplementation reducing fasting levels of anandamide and 2-AG without weight loss in pre-menopausal women with obesity on a weight-loss diet

**DOI:** 10.1186/s13063-020-04586-7

**Published:** 2020-07-17

**Authors:** Fatemeh Haidari, Vahideh Aghamohammadi, Majid Mohammadshahi, Kambiz Ahmadi-Angali, Mohammad Asghari-Jafarabadi

**Affiliations:** 1 Department of Nutrition, Nutrition and Metabolic Research Center, Faculty of Paramedical Sciences, Ahvaz Jundishapur University of Medical Sciences, Golestan Street, Ahvaz, Iran; 2 Department of Nutrition, Khalkhal University of Medical Sciences, Khalkhal, Iran; 3 Department of Statistics and Epidemiology, Faculty of Public Health, Ahvaz Jundishapur University of Medical Sciences, Ahvaz, Iran; 4 Department of Statistics and Epidemiology, Faculty of Health, Tabriz University of Medical Sciences, Tabriz, Iran

**Keywords:** Endocannabinoids, Hypocaloric diet, Obesity, Weight loss, Whey protein

## Abstract

**Background:**

Despite the importance of dairy proteins in modifying of metabolic abnormalities, no attention has been given to their effects on endocannabinoids.

**Methods:**

A total number of 60 obese women were recruited in a 2-month randomized clinical trial. Following random allocation, they were assigned to one of the two groups: control (*n* = 30) and intervention (*n* = 30). Then, all the subjects followed a hypocaloric diet of 800 kcal below estimated energy needs. The intervention group received isocaloric weight-loss diet and whey protein powders (30 g/day). Baseline and 2-month fasting anthropometric, blood glucose, serum insulin, insulin resistance, lipid profile, AEA, and 2-AG were measured.

**Results:**

The study groups were homogenous in terms of baseline characteristics (*p* > 0.05) except for MUFA intake (*p* = 0.021). There were no significant differences in energy and macronutrient intakes in the intervention group compared to the control group at the end of the study (*p* > 0.05). The results of the ANCOVA did not show significant reductions in body weight and BMI of the intervention group compared to the control group (*p* > 0.05); however, WC, body fat, FBS, AEA, 2-AG, total cholesterol, and triglyceride decreased and HDL-c significantly increased in the intervention group compared to the control group (*p* < 0.05).

**Conclusions:**

In this study, the effects of simultaneous weight-loss diet and whey protein supplementation on the reduction of endocannabinoids were determined.

**Trial registration:**

Iranian Registry of Clinical Trials IRCT2017021410181N8. Registered on March 2017.

## Background

Obesity refers to excessive accumulation of fat in the body which can lead to an increase in disability and mortality by predisposing people to other diseases. Ng et al. estimated that in 2013, more than 2 billion people in the world were overweight or obese and about 671 million of them were obese [1]. In Iran, obesity and overweight in recent years are on a growth, and according to a review study in Iran, 21% of people over 18 are obese [2]. Women are also at greater risk for obesity, so that 22.5% of women are obese versus 10.5% of men [3]. Moreover, obesity and overweight are associated with diabetes, myocardial infarction, high blood pressure, dyslipidemia, sleep apnea, gallbladder disease, coronary artery disease, gout, cancer and asthma, mental disorders, and depression [4, 5].

Recently, it has been discovered that ECS (endocannabinoid system) plays a critical role in obesity, high fasting glucose and insulin levels, and insulin resistance and dyslipidemia [6, 7]. The ECS is important in the control and regulation of body weight, since stimulation of CB1 (cannabinoid receptor 1) by anandamide (AEA) and 2-arachidonoylglyceride (2-AG) is associated with increased appetite, higher food intakes, weight gain, and obesity [8]. AEA and 2-AG also play an important role in regulating energy hemostasis, metabolism, and body composition. Obese individuals have higher plasma levels of AEA and 2-AG compared to non-obese controls of both genders [7, 9]. However, plasma endocannabinoids (ECs) are higher in obese women compared to obese men [10, 11]. Sipe et al. verified that circulating ECs could predict obesity risks and proposed targeting ECs as a novel strategy for treating obesity [12].

Whey protein is considered as one of the highest-quality proteins given its amino acid content (high essential and branched-chain amino acid content) and rapid digestibility. Amino acids and peptides from whey protein may also reduce the food intake via release of gut hormones (CCK and GLP-1) [13], and reduction of neuropeptide orexigenic (NPY) and increase of neuropeptide anorexigenic (POMC) in the hypothalamus [14]. The effect of whey on reducing AEA and 2-AG endocannabinoids may be due to the effect on digestive hormones that have a gut-brainstem relationship. These hormones probably block the depolarization-induced suppression by inhibiting voltage-gated calcium channels, resulting in decreased calcium influx and consequently lowered ECB synthesis [15, 16]. CB1 is present in neurons of the enteric nervous system and sensory terminals of vagal and spinal neurons in the gastrointestinal tract. ECS has been found to regulate food intake through the vagus nerve, which connects the medulla and brainstem nuclei associated with satiety with the gastrointestinal tract to monitor the status of digestive processes [6].

Given the putative role of whey protein, plasma EC association with visceral obesity and metabolic profile, and due to the fact that the effect of whey protein on EC concentration is not clear [17], we aimed to evaluate the effects of whey protein supplementation on fasting levels of endocannabinoids, lipid profile, glucose, insulin, HOMA-IR, and anthropometric indices in obese women on a weight-loss diet.

## Methods

### Study design and intervention

The present study was a randomized clinical trial determining the effects of whey supplementation on fasting levels of ECs, lipid profile, glucose, insulin, HOMA-IR, and anthropometric indices in 60 non-menopausal obese women on a weight-loss diet. This research study was registered in the Iranian Registry of Clinical Trials (IRCT registration number: IRCT2017021410181N8).

By referring to dietary clinics in the city of Ahvaz, subjects meeting the inclusion criteria were recruited in this trial after obtaining their written consent (May 2017 to July 2017). The inclusion criteria in this study were as follows: age of 18 years and older, BMI range of 30 to 40 kg/m^2^, absence of menopause, lactation, pregnancy, and food allergies; having no cancer, as well as hepatic, renal, thyroid, and gastrointestinal disorders; no surgery for weight loss; no weight loss over the past 6 months; and taking no herbs and drugs reducing appetite and weight as well as vitamin-mineral supplements.

The sample size was considered according to the changes in AEA level, in response to weight loss, based on the study carried out by Mallipedhi et al., and then, 30 subjects were recruited for each group (*α* = 0.05, *β* = 0.2, S1 = 0.042, S2 = 0.049, μ1 = 0.297, μ2 = 0.209) [18]. The subjects were randomly stratified according to age and BMI using permuted block randomization procedure by Random Allocation Software (RAS), and assigned to one of the two study groups: control (standard weight-loss group, *n* = 30) and intervention (whey supplementation weight-loss group, *n* = 30).

The demographic and physical activity information was also collected through a questionnaire. In order to assess the physical activity of individuals, at the baseline and the end of the study, the International Physical Activity Questionnaire (IPAQ) [19] was used via interviewing and the results were expressed as “high,” “moderate,” and “low” activity.

At the baseline, the 4th week, and the end of study, body weight and height were measured using a seca scale with an accuracy of 100 g and a seca stadiometer with an accuracy of 0.5 cm, respectively, and then, BMI was calculated as body weight (kg) divided by the square of height (m). At the baseline and at the end of study, waist circumference (WC) was measured in standing position using a tape with an accuracy of 1.0 cm at above the iliac crest, just below the lowest rib margin and at the end of normal expiration. TANITA BC-418 body composition analyzer was also employed to calculate total body fat and fat-free mass percentage.

**Fig. 1:**
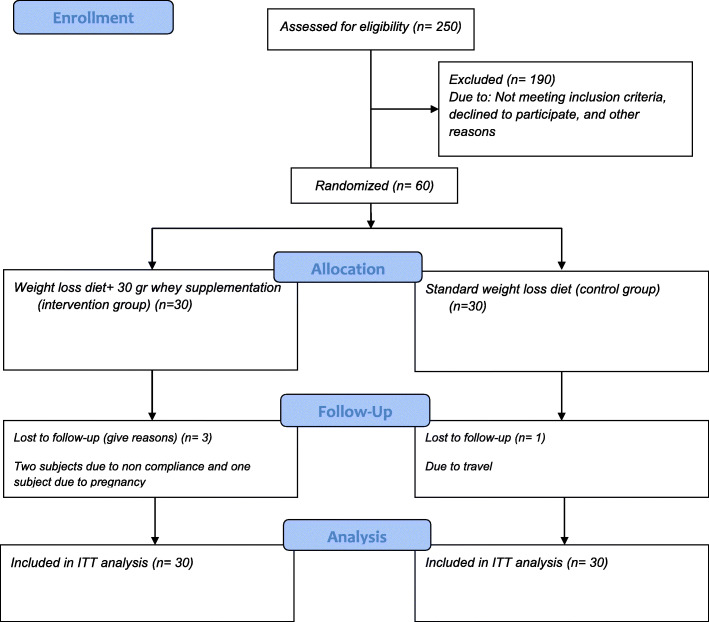
CONSORT flow diagram. We conducted a 2-month open-label, parallel-group, randomized controlled trial to determine the effect of whey protein supplementation on levels of endocannabinoids, glycemic and lipid profile, and body composition in 60 pre-menopausal obese women on a weight-loss diet

Energy requirements were then estimated by the Mifflin Jeor St equation [20], and all subjects followed a hypocaloric diet of 800 kcal below estimated energy needs, which was designed by a trained dietitian in order to reach weight loss of more than 10%. In the control group, the percentage of macronutrients was determined by 55%, 30%, and 15% for carbohydrate, fat, and protein, respectively. The intervention group also received isocaloric weight-loss diet and whey protein powders. Each intervention group subject consumed 30-g whey protein powders daily in the evenings lasting 2 months. The supplements were provided in a sachet form (each sachet containing 30 g of whey protein). Each sachet contained 116 kcal, 0.5 g of lipid, 0.4 g of carbohydrate, and 27.5 g of protein. Whey protein powders were supplied by Karen Pharma & Food Supplement Co., Tehran, Iran. Regarding the calorie of each whey sachet (116 kcal), 912 cal below estimated energy needs was considered for the intervention group. To ensure that the intervention group subjects could regularly consume the supplement, they were contacted every 3 days by a dietitian, or if it was not possible to call them, they were tracked through SMS. Dietary intake was also evaluated by 3-day 24-h recall questionnaires (2 week days and 1 weekend day) at the baseline and at the end of the study. Total energy and macronutrient intake were then calculated using Nut IV (the Hearst Corporation, San Bruno, CA). For the purpose of biochemical evaluation, the 10-cc fasting blood samples were collected at the beginning and at the end of the study. The CONSORT flow diagram of this trial is shown in Fig. 1.

### Biochemical analyses

To determine the plasma AEA and 2-AG, the blood in EDTA-coated tubes was centrifuged at 1500*g* at 4 °C for 15 min and was then stored at − 80 °C. ELISA kits (HANGZHOU EASTBIOPHARM CO., LTD.) were also used to measure levels of endocannabinoids. Blood glucose, total cholesterol, LDL-c, HDL-c, and triglyceride were also determined by enzymatic method with kits from Pars-Azmoon (Tehran, Iran). Insulin levels were similarly measured by chemiluminescent immunoassay. Homeostasis model assessment-insulin resistance (HOMA-IR) was calculated by the following formula: fasting glucose (mg/dl) × fasting insulin (μu/ml)/405.

### Statistical analyses and covariates

All statistical analyses were performed using IBM SPSS Statistics software (version 24) (IBM SPSS Statistics, Armonk, USA). The normality of the variables was confirmed using the Kolmogorov-Smirnov test. To compare the categorical data between treatment groups at the baseline, chi-square test was employed. Independent sample *t* test and paired sample *t* test were applied to compare parametric continuous data between and within the groups, respectively. The Mann-Whitney *U* test and Wilcoxon signed-rank test were also applied to test the differences in asymmetric variables between and within the groups, respectively. To control the confounding variables, analysis of covariance (ANCOVA) test was used in order to determine the differences between the two groups at the post-intervention stage, while adjusting for changes in intake of energy, percentage of carbohydrate, protein, total fat, saturated fatty acid (SFA), monounsaturated fatty acid (MUFA), polyunsaturated fatty acid (PUFA), and other fat and baseline values.

A *p* value less than 0.05 was considered to be statistically significant. All the categorical and numeric variables were presented as number (percentage) and mean (standard deviation), respectively.

## Results

Three subjects in the intervention group (two subjects due to non-compliance and one subject following pregnancy) and one participant in the control group due to travel were lost to follow-up. All the analyses were performed using the intention-to-treat principle (control group, *n* = 30, and intervention group, *n* = 30). The study groups were homogenous in terms of the baseline physical activity levels, demographic characteristics, anthropometric feature, and dietary intake, except for the MUFA intake (*p* > 0.05) (Table 1). No significant changes were also seen in subjects’ physical activity levels during the trial, within or between the two groups (*p* > 0.05).

**Table 1:** Baseline characteristics of study participants

**Variables**	**All subjects**	**Intervention group (*n*** **= 30)**	**Control group (*n*** **= 30)**	***p*value**^**c**^
Age (years)^a^	31.61 (5.73)	31 (6.28)	32.23 (5.17)	0.410
Weight (kg)^a^	89.39 (9.64)	90.03 (10.51)	88.74 (8.81)	0.442
Height (cm)^a^	163.34 (3.57)	163.7 (3.67)	162.98 (3.49)	0.610
BMI (kg/m^2^)^a^	33.45 (2.89)	33.54 (3.17)	33.36 (2.63)	0.820
WC (cm)^a^	99.37 (8.76)	99.63 (8.78)	99.11 (8.88)	0.822
Married status^b^				0.779
Married	42 (70%)	20 (66.66%)	22 (73.33%)	
Unmarried	18 (30%)	10 (33.33%)	8 (26.66%)	
Job status^b^				0.943
Employees	11 (18.3%)	6 (20%)	5 (16.66%)	
Non-administrative employees	10 (16.7)	5 (16.66%)	5 (16.66%)	
Housewife	39 (65)	19 (63.32%)	20 (66.66%)	
Physical activity^b^				1.000
Low		24 (80%)	24 (80%)	
Moderate		6 (20%)	6 (20%)	
High		0 (0%)	0 (0%)	

*BMI* body mass index, *WC* waist circumference

^a^Mean (SD)

^b^Number (%)

^c^Independent *t* test for numeric variables and Pearson’s chi-square test for categorical variables

### Dietary intake

The mean scores of subjects’ dietary intakes are illustrated in Table 2. There were no significant differences in energy and macronutrient intakes (*p* > 0.05), except for the MUFA intake (*p* = 0.027), among the study groups, at the baseline and the end of the study. In the intervention group, compared to the baseline, energy and carbohydrate percentage intakes decreased by 955.26 kcal and 11.67%, respectively. The protein and fat percentage intake increased by 2.53% and 9.53%, respectively. In the control group, compared to the baseline, energy and carbohydrate percentage intakes decreased by 967.05 kcal and 8.96%, respectively, and the protein and fat percentage intake increased by 1.13% and 7.83%, respectively.

**Table 2:** Daily dietary intakes of the study participants at baseline and 2 months after the intervention

**Variable**		**Intervention group (*n*** **= 30)**	**Control group (*n*** **= 30)**	***p*value**^**b,c**^
**Energy (kcal/day)**	Before	2584.61 (208.08)^a^	2552.04 (206.68)	0.545^b^
	After	1629.35 (194.78)	1584.99 (203.23)	0.392^c^
	*p*^d^	**< 0.001**	**< 0.001**	
**Carbohydrate (% energy)**	Before	66.73 (7.58)	64.13 (8.74)	0.224^b^
	After	55.04 (0.99)	55.17 (0.95)	0.612^c^
	*p*^d^	**< 0.001**	**< 0.001**	
**Protein (% energy)**	Before	12.66 (2.97)	13.83 (2.50)	0.116^b^
	After	14.82 (0.53)	14.96 (0.61)	0.350^c^
	*p*^d^	**0.001**	**0.023**	
**Total fat (% energy)**	Before	20.16 (6.3)	22.03 (8.74)	0.465^b^
	After	30.13 (1.10)	29.86 (1.13)	0.359^c^
	*p*^d^	**< 0.001**	**< 0.001**	
**SFA (g)**	Before	18.12 (7.09)	19.39 (9.99)	0.573^b^
	After	13.61 (4.30)	14.26 (6.31)	0.646^c^
	*p*^d^	**< 0.001**	**< 0.001**	
**MUFA (g)**	Before	13.41 (5.94)	18.61 (1.96)	**0.027**^b^
	After	7.44 (3.66)	10.25 (7.16)	**0.062**^c^
	*p*^d^	**< 0.001**	**< 0.001**	
**PUFA (g)**	Before	12.28 (8.95)	12.99 (10.03)	0.771^b^
	After	25.00 (10.91)	21.84 (10.24)	0.249^c^
	*p*^d^	**< 0.001**	**0.013**	
**Other fat (g)**	Before	15.73 (11.90)	12.76 (4.70)	0.208^b^
	After	8.46 (7.42)	6.31 (2.97)	0.149^c^
	*p*^d^	**< 0.001**	**< 0.001**	

*MD* mean difference, *CI* confidence interval, *SFA* saturated fatty acid, *MUFA* monounsaturated fatty acid, *PUFA* polyunsaturated fatty acid

^a^Mean (SD). *p* values of statistical significance (*p* < 0.05) are presented in bold

^b^Independent *t* test for intake of energy, carbohydrate, protein, and total fat and Mann-Whitney *U* for SFA, MUFA, PUFA, and other fat

^c^Analysis of covariance (adjusted for changes in intake of energy, percent of carbohydrate, protein, total fat, SFA, MUFA, PUFA, and other fat and baseline values)

^d^Paired *t* test intake of energy, carbohydrate, protein, and total fat and Wilcoxon for SFA, MUFA, PUFA, and other fat

### Anthropometric measurements

As shown in Table 3, there were no significant differences in anthropometric measurements between the study groups at the baseline (*p* > 0.05). At the end of the study, all the anthropometric indices significantly reduced in both groups (*p* < 0. 001). The results of ANCOVA did not show significant reductions in body weight and BMI in the intervention group compared to those in the control group (*p* > 0.05), but WC and body fat were decreased significantly in the intervention group compared to those in the control group. Regarding the fat-free mass, it decreased significantly in the control group (− 2.02, CI − 2.36 to − 1.69) compared to those in the intervention group (− 1.00, CI − 1.38 to − 0.63).

**Table 3:** Anthropometric indices of study groups at baseline and end of the intervention

**Variable**		**Intervention group (*n*** **= 30)**	**Control group (*n*** **= 30)**	***p*value**^**b,c**^
**Body weight (kg)**	Before	90.03 (10.51)^a^	88.74 (8.81)	610^b^
	After	86.22 (9.27)	85.55 (8.83)	0.645^c^
	*p*^d^	**< 0.001**	**< 0.001**	
**BMI (kg/m**^**2**^**)**	Before	33.54 (3.17)	33.36 (2.63)	0.820^b^
	After	32.14 (2.9)	32.16 (2.61)	0.611^c^
	*p*^d^	**< 0.001**	**< 0.001**	
**WC (cm)**	Before	99.63 (8.78)	99.11 (8.88)	0.822^b^
	After	95.60 (8.48)	97.52 (8.80)	**< 0.001**^c^
	*p*^d^	**< 0.001**	**< 0.001**	
**Body fat (kg)**	Before	39.16 (7.12)	38.35 (6.26)	0.640^b^
	After	34.81 (5.98)	35.38 (5.89)	**0 < 001**^c^
	*p*^d^	**< 0.001**	**< 0.001**	
**Body fat-free mass (%)**	Before	22.08 (2.24)	22.30 (1.86)	0.697^b^
	After	21.07 (1.90)	20.25 (1.82)	**0.002**^c^
	*p*^d^	**< 0.001**	**< 0.001**	

*MD* mean difference, *CI* confidence interval, *BMI* body mass index, *WC* waist circumference

^a^Mean (SD). *p* values of statistical significance (*p* < 0.05) are presented in bold

^b^Independent *t* test

^c^Analysis of covariance (adjusted for changes in intake of energy, percent of carbohydrate, protein, total fat, SFA, MUFA, PUFA, and other fat and baseline values)

^d^Paired *t* test

### Biochemical parameters

No significant between-group differences were found in the biochemical values, at the baseline (*p* > 0.05). At the end of the study, the AEA and 2-AG levels significantly decreased in the whey intervention group (− 6.02 ng/ml (CI − 8.75 to − 3.28), < 0.001; − 8.28 ng/ml (CI − 12.00 to − 4.54), < 0.001, respectively). The mean difference in the AEA and 2-AG levels between the two groups after the intervention was significant (− 1.76 (CI − 4.42 to 0.90), *p* value < 0.001; − 2.52 (CI − 6.71 to 1.67), *p* value< 0.001, respectively) (tested by ANCOVA after adjusting for covariates).

As shown in Table 4, total cholesterol, LDL-c, and triglyceride levels significantly reduced and HDL-c levels increased in the intervention group compared to baseline (*p* < 0.05), but changes in these variables were not significant in the control group (*p* > 0.05). Fasting insulin levels and HOMA-IR also reduced significantly in both groups at the end of the study (*p* < 0.05). In the intervention group, levels of fasting glucose, total cholesterol, and triglyceride significantly decreased (*p* = 0.044, *p* = 0.013, *p* = 0.03, respectively), and HDL-c (*p* = 0.006) significantly increased compared to the control group (tested by ANCOVA after adjusting for covariates) (Table 4).

**Table 4:** Biochemical parameter values of the study groups at baseline and at the end of the intervention

**Variable**		**Intervention group (*n*** **= 30)**	**Control group (*n*** **= 30)**	***p*value**^**b,c**^
**AEA (ng/ml)**	Before	11.50 (8.83)^a^	8.07 (6.21)	0.090^b^
	After	5.49 (4.23)	7.25 (5.90)	**< 0.001**^c^
	*p*^d^	**< 0.001**	**0.006**	
**2-AG (ng/ml)**	Before	16.39 (13.36)	11.92 (8.99)	0.183^b^
	After	8.11 (7.72)	10.64 (8.38)	**0.001**^c^
	*p*^d^	**< 0.001**	**0.012**	
**TC (mg/dl)**	Before	141.51 (20.14)	148.53 (26.37)	0.257^b^
	After	129.63 (19.72)	146.79 (24.60)	**0.013**^c^
	*p*^d^	**0.003**	0.707	
**LDL-C (mg/dl)**	Before	75.3 (15.98)	75.06 (12.53)	0.950^b^
	After	68.46 (9.05)	72.51 (11.07)	0.095^c^
	*p*^d^	**0.014**	0.258	
**HDL-C (mg/dl)**	Before	40.33 (4.03)	41.43 (5.36)	0.373b
	After	44.21 (4.71)	41.41 (5.60)	**0.006**^c^
	*p*^d^	**< 0.001**	0.983	
**TG (mg/dl)**	Before	132.03 (34.33)	136.96 (50.83)	0.661^b^
	After	108.49 (25.18)	130.17 (49.98)	**0.030**^c^
	*p*^d^	**< 0.001**	0.346	
**FBS (mg/dl)**	Before	86.66 (9.89)	81.76 (10.09)	0.062^b^
	After	79.37 (8.31)	79.39 (8.08)	**0.044**^c^
	*p*^d^	**< 0.001**	**0.035**	
**Insulin (μIU/ml)**	Before	9.30 (5.10)	7.62 (5.16)	0.220^b^
	After	5.65 (2.33)	5.91 (3.20)	0.124^c^
	*p*^d^	**< 0.001**	**0.018**	
**HOMA-IR**	Before	1.96 (1.00)	1.60 (1.24)	0.213^b^
	After	1.11 (0.49)	1.17 (0.69)	0.070^c^
	*p*^d^	**< 0.001**	**0.010**	

*MD* mean difference, *CI* confidence interval, *AEA* anandamide (AEA), *2-AG* 2-arachidonoylglyceride, *TC* total cholesterol, *LDL-C* low-density lipoprotein cholesterol, *HDL-C* high-density lipoprotein cholesterol, *TG* triglycerides, *FBS* fasting blood sugar, *HOMA-IR* homeostasis model assessment for insulin resistance

^a^Mean (SD). *p* values of statistical significance (*p* < 0.05) are presented in bold

^b^Independent *t* test for TC, HDL-c, LDL-c, TG, and FBS and Mann-Whitney *U* for AEA, 2-AG, insulin, and HOMA-IR

^c^Analysis of covariance (adjusted for changes in intake of energy, percent of carbohydrate, protein, total fat, SFA, MUFA, PUFA, and other fat and baseline values)

^d^Paired *t* test for TC, HDL-c, LDL-c, TG, and FBS and Wilcoxon for AEA, 2-AG, insulin, and HOMA-IR

## Discussion

The present trial evaluated the effect of whey protein supplementation on fasting levels of endocannabinoids, lipid profile, glucose, insulin, HOMA-IR, and anthropometric indices in obese women on a weight-loss diet. Accordingly, the whey supplementation along with weight-loss diet induced a significant decrease of AEA and 2-AG levels in the intervention group compared to the control group.

The regulation of ECS is impaired in obesity, and increased levels of endocannabinoids in obesity lead to increased fatty cell size, fatty tissue macrophage content, inflammatory processes, and metabolic changes associated with obesity [21].

In human studies, the impact of weight loss from hypocaloric diets on ECs is conflicting. In this respect, Engeli et al. quantified serum AEA, 2-AG levels, CB1, and FAAH gene expression in obese postmenopausal women and observed no changes after 5% weight loss from caloric restriction [22]. Also, it was reported that at least 10% weight loss was required to be achieved to influence weight loss on circulating AEA and 2-AG levels [22, 23]. In contrast, one of the important findings of the present study was that supplementation of whey protein along with weight-loss diet could reduce AEA and 2-AG without significant drop in weight and BMI compared with the control group which suggested that whey protein could improve obesity biomarkers through the mechanisms other than weight loss. In this study, weight loss for both groups was less than 5%. It should be noted that the study population and the weight-loss method were different from those in other investigations [7, 18, 22, 23].

Recent studies have demonstrated the significant role of whey protein supplementation in glycemia control, possibly through the stimulation of incretin hormones, which increase fasting and postprandial insulin release and improve insulin sensitivity [24]. In the other studies, consistent with the present study, whey protein could decrease the blood glucose, but these studies considered non-obese subjects (with BMI 26.2 to 28.2) [25, 26]. In a study by Sukkar et al. after 10 days of protein-sparing modified fast followed by 20 days of a low-calorie diet, in patients with morbid obesity, fasting glucose, insulin, and HOMA-IR were significantly reduced [27]. High BMI could also have a negative effect on the response of incretins to whey supplementation, and it is unclear how diabetes and obesity reduce the response of incretins to whey supplementation. It has been shown that in obese women with metabolic syndrome, insulin resistance can have a significant positive correlation with plasma levels of branched-chain amino acids, but weight loss could eliminate this correlation [28]. Almario et al. in a study assessing the effect of whey on glycemic indices in diabetic patients suggested that lower BMI, normal triglycerides, and low serum GLP-1 were glucose-response positive predictive variables in response to supplementation of whey protein [29]. The bioactive peptides were identified in whey protein which might also serve as endogenous inhibitors of dipeptidyl peptidase-4 (DPP-4) in the proximal gut, and prevent incretin degradation [14, 24].

In this study, after whey isolate supplementation along with weight-loss diet, significant decrease of total cholesterol and triglyceride levels and increase of HDL-C levels were observed. One of the main components of whey is β-lactoglobulin (45–57%) that has higher content of branched-chain amino acids (~ 25.1%), captures hydrophobic molecules, and then contributes to the reduction of intestinal absorption of lipids [14]. Whey protein supplementation could also improve lipid profiles and metabolic syndromes by increasing the expression of cholesterol carriers and decreasing cholesterol accumulation and expression of lipogenic lipid enzymes [30]. Whey protein consists of angiotensin converting enzyme (ACE) inhibitors. Angiotensin 2 causes an increased expression of the fatty acid synthase enzyme; therefore, the inhibition of ACE by whey protein results in decreased endogenous production of lipids [31]. Pal et al. demonstrated that 12 weeks of supplementation with whey proteins in comparison to casein and glucose (control) supplementation could improve fasting lipids and insulin levels in overweight and obese individuals [32]. Hong et al. also examined anti-obesity effects of whey in rats with high-fat diet and eventually observed lower levels of total cholesterol, triglyceride, and LDL-c in the whey-recipient group compared to other study groups [33]. Moreover, Sukkar et al. after 10 days of whey protein-sparing modified fasting (Promofast) observed a significant improvement in lipid profile [27].

At the end of the study, all the anthropometric indices significantly reduced in both groups. The results of ANCOVA did not show significant reductions in body weight and BMI in the intervention group compared to those in the control group. The anorexic effect of whey protein is observed prevalently when whey protein is assumed on fasting in rats and humans [13, 34, 35] because whey protein is a fast protein [13] but only in fasting conditions. Moreover, Soop et al. showed if whey protein is mixed in a meal with casein, casein exhibits a more sustained effect on plasma amino acid levels and muscle protein accretion compared with WP and therefore cannot inhibit appetite [36]. So, the scarce weight effect in the present study could be explained that the whey supplementation was not targeted to reduce appetite but only to verify the efficacy of WP on endocannabinoids.

According to the findings of this study, a supplement of 30 g of protein for 2 months in non-menopausal women under a weight-loss diet resulted in a significant reduction in body fat mass and waist circumference, as well as a lower loss mass muscle compared to the control group. Frestedt et al. evaluated the effects of 40 g of whey along with a weight-loss diet on body composition for 12 weeks; in this study, the whey-receiving group had the greater reduction in body fat and lower decrease in muscle mass compared to maltodextrin. Whey supplementation also led to 6.1% reduction in body fat mass, while a 5% reduction in body fat could reduce the risk of obesity-related diseases [37]. Also, in the present study, 7.14% reduction in body fat mass was observed in the whey protein group. Whey protein supplement can reduce body fat through hormones that affect the metabolism, such as reducing the uptake of the T3 hormone [38]. In contrast, Aldrich et al. rejected the hypothesis that a whey protein intervention along with weight-loss diet would result in greater weight loss and improve body composition compared to standard weight-loss diets, but significant differences in regional fat loss and in decreased blood pressure were observed in the whey protein group [39].

Differences in delivery methods (i.e., preload, as a stand-alone supplement, with a meal), varying doses and forms of whey protein (i.e., WPC, WPI, WPH), duration of trial, sample size, and the population under study yielded the contradictory results.

Despite roles of dairy proteins in modulating inflammation, oxidative stress, hypertension, risk factors of metabolic diseases, food intake behaviors, and other processes, no attention had been given to their effect on AEA, 2-AG, and other endocannabinoids. The ECs might also represent a key target for the treatment of abdominal obesity and associated metabolic changes. It seems that ECB is a key player in macronutrient metabolism between organs involved in food intake and systemic energy balance [17]. Among the strengths of the study was the use of a lower dose of whey protein, regular follow-up of subjects for observing diet, supplement intake and physical activity, and assessment of effect of whey protein on endocannabinoid levels for the first time. One of the limitations of this study was that CB1 and ECs metabolizing enzyme gene expression were not measured. Another limitation was using the ELISA kits instead HPLC to measure plasma endocannabinoids. Further researches are needed to determine the effect of whey protein on CB1 and ECs metabolizing enzyme gene expression and levels of other endocannabinoids.

## Conclusions

The effects of simultaneous weight-loss diet and whey protein supplementation on the reduction of anandamide and 2-arachidonoylglyceride were demonstrated in this study. The other benefits of whey protein supplementation along with weight-loss diet on health were reported.

## Data Availability

The data could be available on request to the authors.

## Abbreviations

ECS: Endocannabinoid system
EC: Endocannabinoid
MD: Mean difference
CI: Confidence interval
SFA: Saturated fatty acid
MUFA: Monounsaturated fatty acid
PUFA: Polyunsaturated fatty acid
IPAQ: International Physical Activity Questionnaire
BMI: Body mass index
WC: Waist circumference
AEA: Anandamide
2-AG: 2-Arachidonoylglyceride
TC: Total cholesterol
LDL-C: Low-density lipoprotein cholesterol
HDL-C: High-density lipoprotein cholesterol
TG: Triglycerides
FBS: Fasting blood sugar
HOMA-IR: Homeostasis model assessment for insulin resistance
